# Quality of life, functions, bradykinesia, and dyskinesia in patients with schizophrenia

**DOI:** 10.1192/j.eurpsy.2026.10509

**Published:** 2026-07-31

**Authors:** S.-M. Wang, H.-C. Lee

**Affiliations:** 1 Department of Occupational Therapy, National Cheng Kung University, Tainan; 2 Research Center of Sleep Medicine, Taipei Medical University, Taipei, Taiwan

## Abstract

**Introduction:**

Movement abnormalities, including bradykinesia (slow movements) and dyskinesia (involuntary muscle contractions), consistently appear in different stages of psychosis continuum. Accumulating evidence supports the notion that movement abnormalities are a cardinal part of schizophrenia and not merely side effects of antipsychotics. Nevertheless, we still have an insufficient understanding of these movement issues in schizophrenia. For example, little research has focused on exploring whether movement abnormalities were significant correlates of quality of life and functions in patients with schizophrenia. Identifying these correlates paves the way for recognizing meaningful intervention targets and developing tailored intervention programs.

**Objectives:**

To examine association between quality of life and functions with bradykinesia and dyskinesia in patients with schizophrenia.

**Methods:**

Currently, we have recruited 13 participants with schizophrenia and 5 healthy participants. Quality of life was assessed using the short version of the World Health Organization Quality of Life questionnaire. Daily living and work functions were assessed using Jebsen-Taylor Hand Function Test and total working hours in the past four weeks respectively. Bradykinesia was assessed using Box and Block Test (specifically assessing upper-limb gross motor speed), Purdue Pegboard Test (assessing upper-limb fine motor speed), and the hypokinesia domain of Extrapyramidal Symptom Rating Scale. Dyskinesia was assessed using Abnormal Involuntary Movement Scale. Spearman’s correlation coefficients were calculated.

**Results:**

For the total sample (N = 18), higher quality of life, a higher daily living function, and a higher work function were related to faster upper-limb gross motor (rho = 0.65, *p* = 0.004; rho = −0.77, *p* < 0.001; rho = 0.73, *p* = 0.001) and fine motor (rho = 0.56, *p* = 0.016; rho = −0.84, *p* < 0.001; rho = 0.68, *p* = 0.002) and less severe hypokinesia (rho = −0.60, *p* = 0.009; rho = 0.83, *p*<0.001; rho = −0.73, *p* = 0.001), but not related to dyskinesia (rho = −0.14, *p* = 0.587; rho = 0.35, *p* = 0.150; rho = −0.38, *p* = 0.120). For participants with schizophrenia (*n* = 13), a higher daily living function was related to faster upper-limb fine motor (rho = −0.68, *p* = 0.011) and less severe hypokinesia (rho = 0.56, *p* = 0.048). The other correlations were not significant.

**Conclusions:**

Bradykinesia was a significant correlate of daily living functions in patients with schizophrenia. Healthcare practitioners should target bradykinesia to develop effective interventions for these patients, thus contributing to enhancing their independent living.

**Disclosure of Interest:**

None Declared

